# Impact of Evoked Compound Action Potential (ECAP)-Controlled Closed-Loop Spinal Cord Stimulation in Refractory Lumbar Radiculopathy: A Case Report

**DOI:** 10.7759/cureus.102799

**Published:** 2026-02-01

**Authors:** Aviraj Soin, Sarah Kassis, Richard J Witte, Ben Sloop, Cameron Brittain

**Affiliations:** 1 Pain Clinic, The Ohio Pain Clinic, Dayton, USA; 2 Department of Medicine, Northeast Ohio Medical University, Dayton, USA; 3 Anesthesiology, Wright State University, Dayton, USA; 4 Medicine and Pain, Elite Pain Doctors, Mason, USA; 5 Osteopathic Medicine, Ohio University College of Medicine, Athens, USA

**Keywords:** closed-loop scs, closed-loop spinal cord stimulation, ecaps controlled closed-loop scs, lumbar radiculopathy, lumbosacral radiculopathy, sciatica

## Abstract

Lumbar radiculopathy is a common pain disorder characterized by radiating pain from the lower back into the lower extremities. Conventional treatment options are frequently employed; however, many patients experience limited or inadequate symptom relief. Evoked Compound Action Potential (ECAP)-controlled closed-loop spinal cord stimulation (SCS) has emerged as a neuromodulation strategy that utilizes real-time physiological feedback to optimize stimulation delivery. This case report describes the use of ECAP-controlled closed-loop SCS in a patient with refractory lumbar radiculopathy. A white male in his 40s presented with chronic, debilitating right-sided lumbar radiculopathy refractory to prior treatments, which was caused by irritation, compression, or inflammation of a spinal nerve root. At baseline, the patient reported a numeric rating scale (NRS) pain score of 7/10, including limited sleep duration (four hours), standing tolerance (10 minutes), and walking tolerance (five minutes).

The patient underwent an ECAPs-assisted closed-loop SCS using a Saluda Medical spinal cord stimulator system with a single 12-electrode lead positioned at the top of the T7 vertebral level. During the trial period (over five days), approximately 24.2 million automated stimulation adjustments were performed, and device utilization was recorded at 100%. Following treatment, the patient reported a significant reduction in pain (NRS=1) and had marked improvement in sleep (6 hours), standing tolerance (four hours), and walking tolerance (one hour). This case demonstrates meaningful and sustained improvement in pain and functional outcomes following ECAP-controlled closed-loop SCS in a patient with refractory lumbar radiculopathy. While these findings are promising, results from a single case should be interpreted cautiously, and further studies are warranted, as clinical outcomes were assessed at the end of a five-day SCS trial period.

## Introduction

Lumbar radiculopathy is a prevalent medical condition associated with greater pain severity, increased healthcare utilization, and higher levels of disability compared with other non-specific low back pain [1]. It is estimated that 40% to 70% of individuals will experience lumbar radiculopathy in their lifetime, contributing to healthcare costs and societal burden [2]. This condition is usually characterized by sharp, shooting pain radiating from the lower back into the lower extremities and may be accompanied by sensory disturbances or motor deficits in severe cases [3].

Conservative management strategies for lumbar radiculopathy, including pharmacological therapy, epidural steroid injections, structured physical rehabilitation, radiofrequency ablation, spinal manipulative therapy, function-specific physical training, specific exercise, and transcutaneous electrical nerve stimulation (TENS) therapy, can be used for the management of lumbar radiculopathy [4-6]. Despite these interventions, a subset of patients remains refractory to treatment, particularly in patients who have failed interventional pain options and are not candidates for surgical management. In such cases, neuromodulation through spinal cord stimulation (SCS) has emerged as a viable therapeutic option [7].

In the field of bioelectronics medicine, SCS offers a well-established, non-pharmacological, and implantable therapy for chronic pain management [8]. Advances in neuromodulation technology, including burst stimulation, dorsal root ganglion stimulation, and high-frequency stimulation, have addressed some limitations of conventional tonic SCS [9]. Traditionally, SCS systems operate in an open-loop configuration, delivering fixed stimulation parameters regardless of physiological variability. However, changes in posture, movement, and cerebrospinal fluid dynamics can alter the distance between electrodes and neural tissue, resulting in variable neural recruitment and inconsistent therapeutic effects [10]. To overcome these limitations, closed-loop SCS systems have been developed.

These systems utilize evoked compound action potentials (ECAPs) as an objective biomarker of spinal cord activation to help guide programming and optimize stimulation accuracy and activation of neurons [11]. It is also helpful in continuously modulating the intensity of stimulation in real-time, ensuring more effective and stable pain management [12]. In addition, ECAPs assisted closed-loop SCS can deliver a varied amount of waveforms, with the adjustments of programmed amplitude according to the comfort of the patients and automatically adjusts up to 50 times/second [13]. It has more sensitivity to the movement of patients and rapidly adjusts stimulations; by these adjustments, the system easily reduces the variability in the dose reaching the spinal cord [13]. Due to these adjustments, it can maintain a predetermined neural recruitment level, thus patients with chronic pain demonstrated improved pain relief [14].

The present case report describes the application of ECAP-controlled closed-loop SCS in a patient with refractory lumbar radiculopathy. This case study is unique in demonstrating effective radicular pain control using a thoracic (T7) lead position combined with real-time ECAP feedback, allowing automatic adjustment of stimulation to maintain consistent neural activation.

## Case presentation

**Figure 1:**
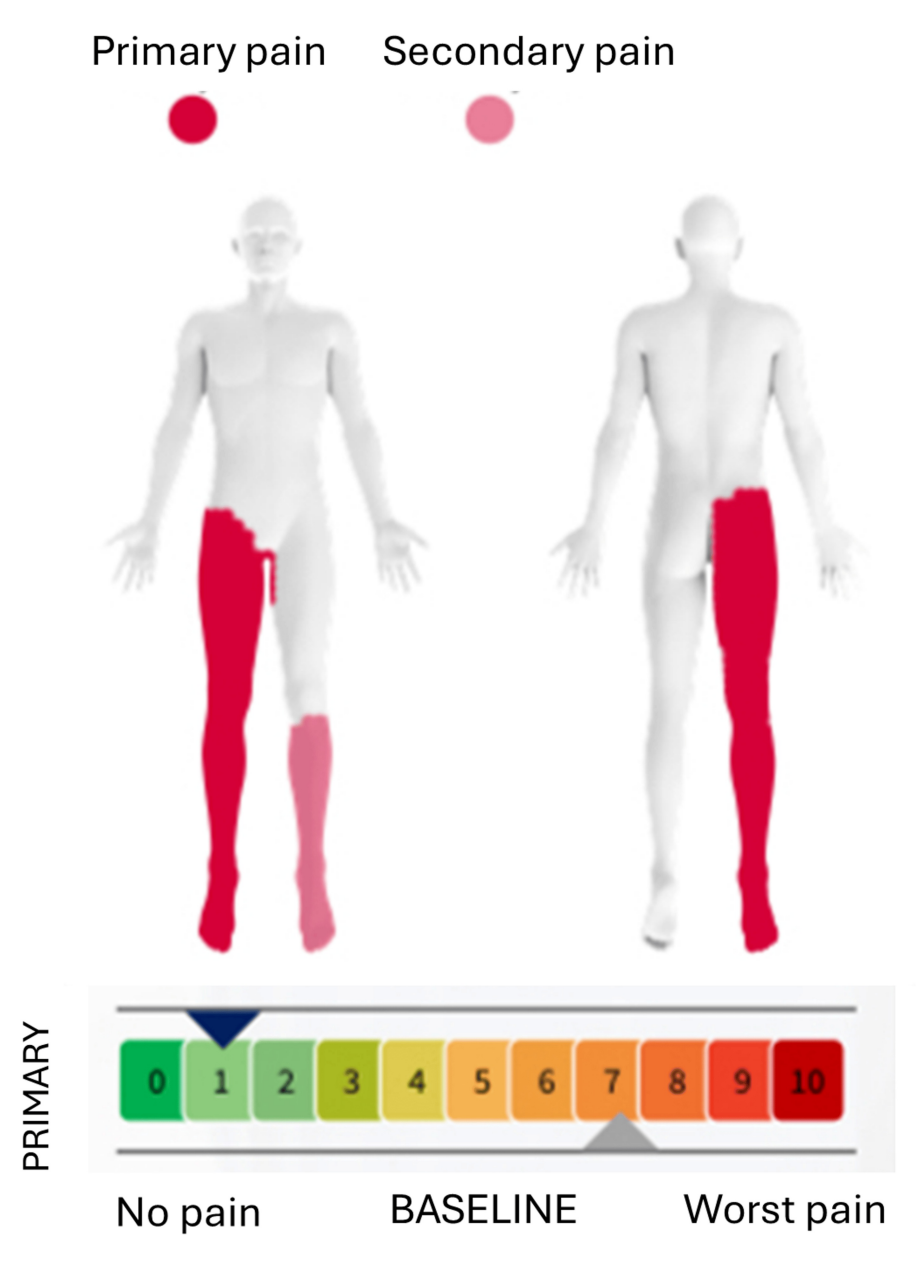
Patient with lumbar radiculopathy, indicating a severe condition according to the NRS scores (0 indicates no pain, 1-3 indicates mild pain, 4-6 indicates moderate pain, 7-10 indicates severe pain). The image is created by the authors.

A white male patient in his 40s presented with chronic lumbar radiculopathy characterized by persistent radiating low back pain in a dermatomal distribution. Clinical history indicated that patient had pain for over six months and faced progression of symptoms, along with physical constrains, heavy lifting. Different treatments, including analgesics, and physiotherapy were used, however treatments were found refractory. Meanwhile, the magnetic resonance imaging (MRI) identified the underlying cause, which was the persistent compression and inflammation of a spinal nerve root in the lower back. Before proceeding further, informed consent was obtained from the patient. The patient’s baseline pain score was a 7/10 according to NRS (0 indicates no pain, 1-3 indicates mild pain, 4-6 indicates moderate pain, 7-10 indicates severe pain) and had several functional limitations, including limited sleep duration (four hours), standing tolerance (10 minutes), and walking tolerance (five minutes). The patient identified three primary therapy goals: improving sleep duration beyond four hours, increasing standing tolerance beyond 10 minutes, and increasing walking tolerance beyond five minutes (Figure 1).

**Figure 2:**
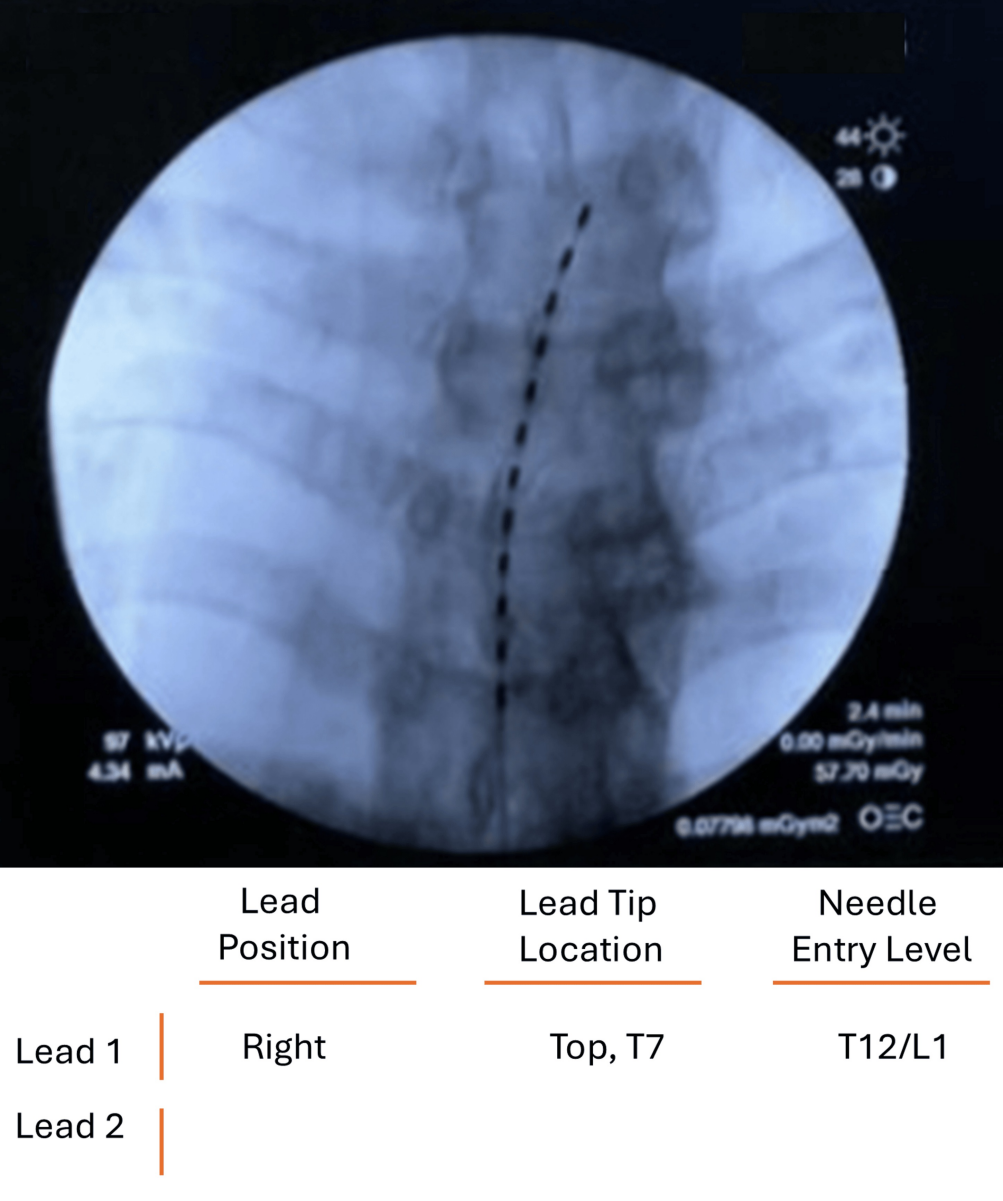
Fluoroscopic imaging of the spinal cord stimulator The image demonstrated a lead spanning the T7 vertebral body.

The patient elected to undergo an SCS trial, as it stimulates the gamma-aminobutyric acid release and reduces the production of glutamate, which is helpful in the reduction of pain. A Saluda Medical ECAP-controlled closed-loop SCS system (Evoke® System, Saluda Medical Pty Ltd, Artarmon, New South Wales, Australia) was implanted using a single 12-electrode lead positioned at the superior aspect of the T7 vertebral level. This was done to achieve optimal dorsal column coverage of the lumbosacral dermatomes through rostrocaudal current spread, a strategy commonly used when targeting lower extremity radicular pain. This level also allowed stable ECAP capture, while minimizing uncomfortable paresthesia and ensuring consistent stimulation during posture changes. Meanwhile, paresthesia mapping was performed intraoperatively to ensure adequate coverage of right-sided lower extremity pain. Fluoroscopic imaging confirmed appropriate lead placement slightly right of the midline plane in order to optimize right-sided radicular coverage (Figure 2).

Stimulation was delivered using the inferior four electrode contacts using a +/-/+ distribution. Programming parameters included a pulse width of 240 microseconds and a stimulation frequency of 40 Hz. The maximum stimulation current was set to 10 mA. The patient sensitivity was 40 microvolts per milliamp. The ECAP target max was 80 microvolts, and the ECAP target increment was 1 microvolt. The ECAP-controlled closed-loop system automatically adjusted stimulation output to maintain consistent neural recruitment throughout the trial period.

**Figure 3:**
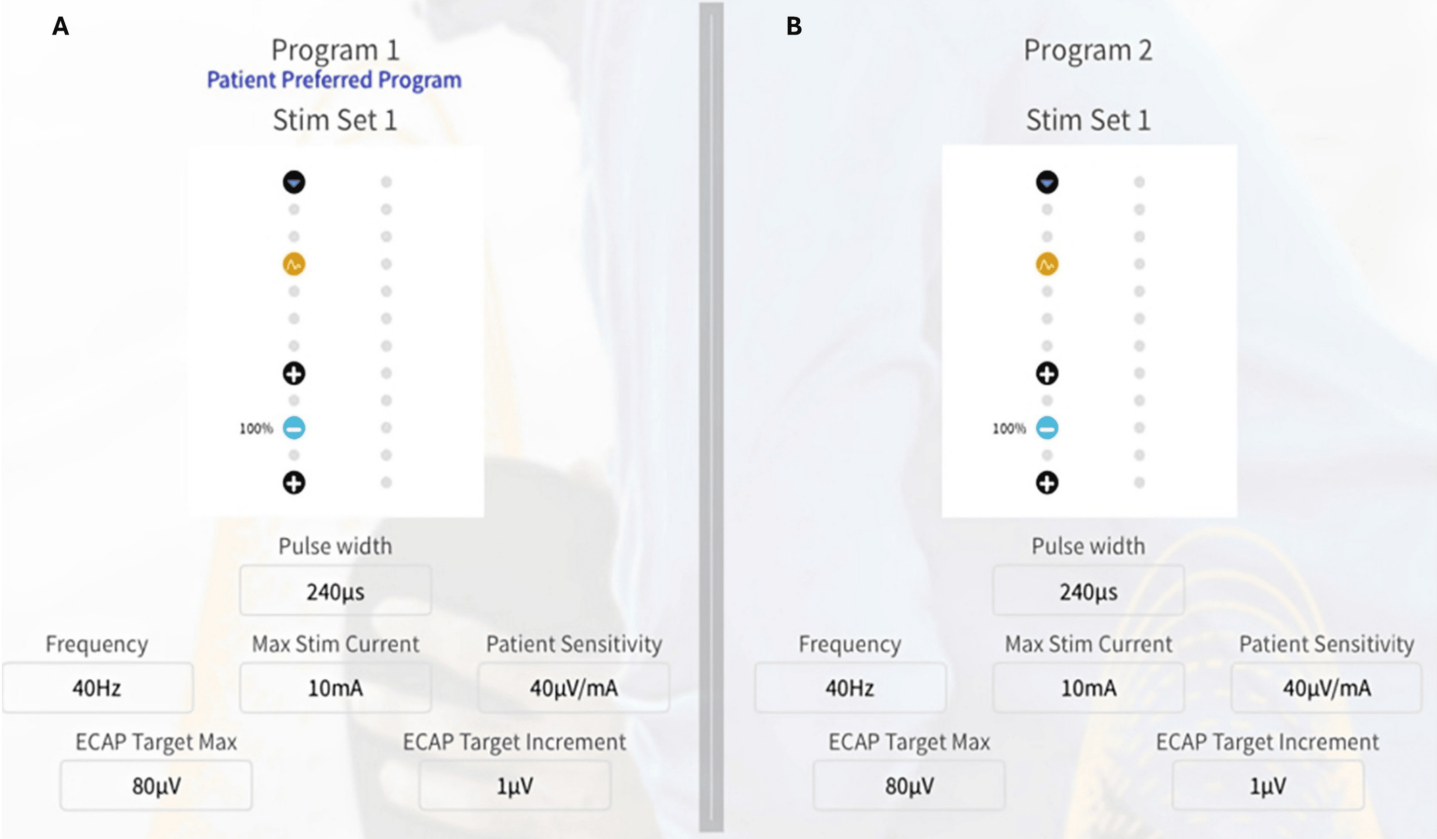
Programming information utilized by the patient with lumbar radiculopathy for his entire treatment period A) Program 1, B) Program 2. ECAP: Evoked Compound Action Potentials. The image is created by the authors.

The SCS trial was performed over five days, and it can maintain superior, long-term pain relief (up to 36 months). The dose response curve demonstrated a consistent relationship between voltage per unit of delivered current (milliamps) and the time to perception level, which was observed at approximately 1.5 milliseconds (Figure 3).

**Figure 4:**
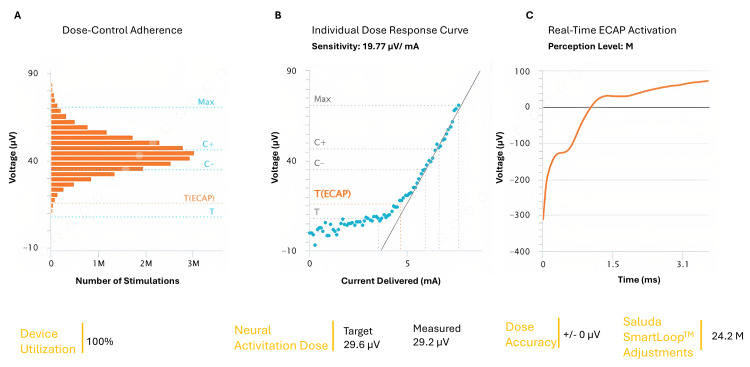
Closed-loop stimulation performance demonstrated highly stable neural activation in a patient with lumbar radiculopathy A) Dose-Control adherence, B) Individual dose response curve, C) Real-time ECAP activation. ECAP: Evoked Compound Action Potentials. The image is created by the authors.

During this time, the system performed approximately 24.2 million automated adjustments, reflecting continuous adaptation to physiological changes. Device utilization during the trial was 100% (Figure 4).

**Figure 5:**
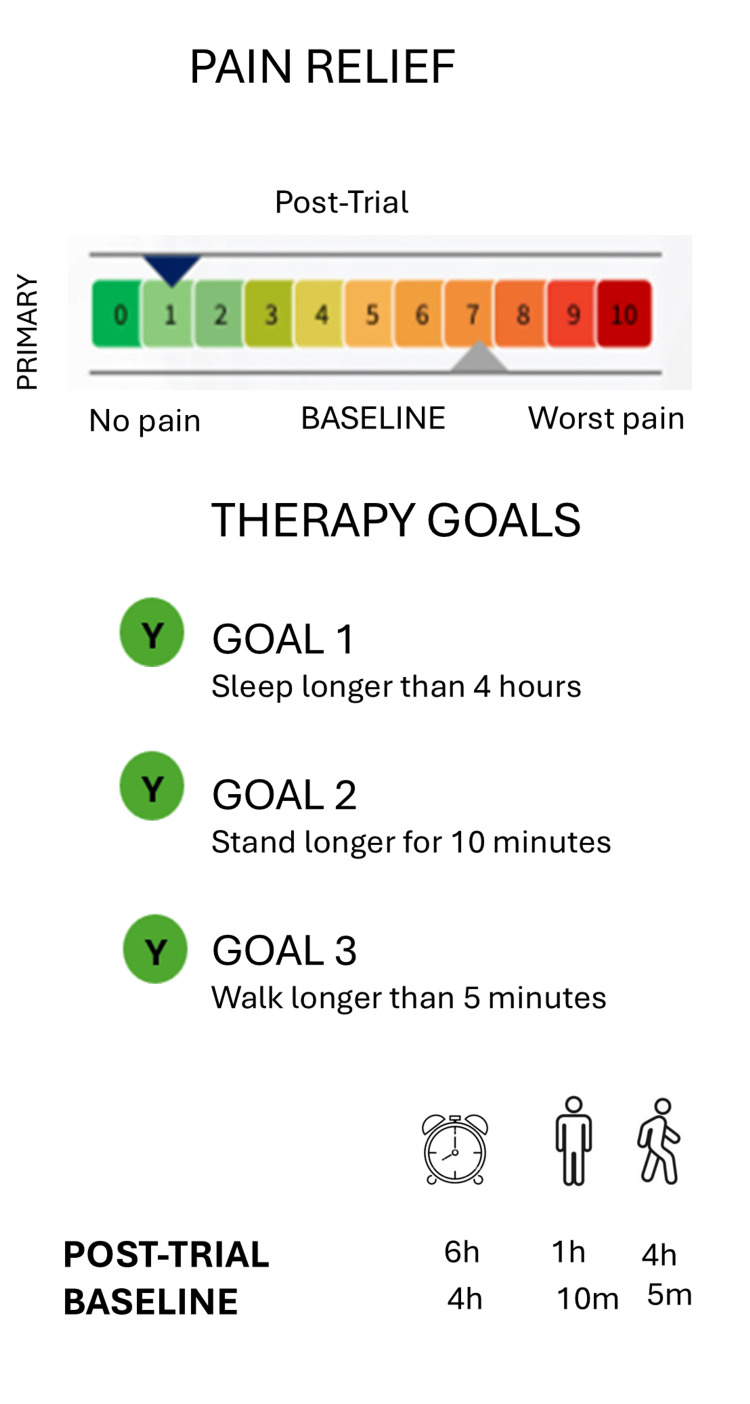
Improvement in the pain and functional outcomes associated with lumbar radiculopathy. The image is created by the authors.

Following treatment, the patient reported substantial improvement across all predefined therapy goals. Pain intensity decreased to an NRS score of 1/10. Sleep duration improved from four to six hours per night. Standing tolerance increased from 10 minutes to approximately one hour, and walking tolerance improved from five minutes to approximately four hours (Figure 5). In addition, other contributing factors, like placebo factors, may also contribute to the reduction of pain.

## Discussion

This case study demonstrates meaningful clinical improvement in pain intensity and functional outcomes following an ECAP-controlled closed-loop SCS in a patient with refractory lumbar radiculopathy. The observed reductions in pain, along with improvements in sleep, standing, and walking tolerance, highlight the potential advantages of closed-loop neuromodulation in managing complex chronic pain conditions. Unlike traditional open-loop SCS, which delivers fixed stimulation regardless of physiological changes, closed-loop SCS continuously adjusts stimulation output based on real-time ECAP feedback from the spinal cord. This allows the system to maintain consistent neural activation despite movement, posture changes, or electrode-tissue impedance shifts, which may control pain and reduce overstimulation or loss of efficacy. This feature makes our case different from previously reported thoracic or lumbar SCS cases that relied on fixed-output systems.

Our findings are consistent with prior reports supporting the efficacy of closed-loop SCS. In a case study by Parker et al., closed-loop SCS was used in a 48-year-old female with chronic refractory pain, in which pain scores dropped from 10 to 2 on the NRS scale [15]. Additionally, a randomized controlled trial comparing ECAP-controlled closed-loop SCS with open-loop SCS in patients with back and leg pain demonstrated superior pain reduction in the closed-loop group after 24 months of follow-up [16]. A systematic review also concluded that novel SCS when compared with conventional SCS had superior back pain relief (novel SCS: -2.34 mean difference (MD), 95% CI, -2.96 to -1.73; conventional SCS: -1.17 MD, 95% CI, -1.64 to -0.70) Likewise novel SCS was found to be effective in reducing intensity of leg pain (novel SCS: -4.01 MD, 95% CI, -2.96 to -1.73; conventional SCS: -1.17 MD, 95% CI, -1.64 to -0.70) [17].

An important observation in this case was the stability of pain relief across multiple functional domains. Closed-loop SCS systems continuously adjust stimulation output to compensate for physiological variability, including changes in posture and activity level. ECAP amplitude reflects the degree of dorsal column fiber recruitment, allowing the system to maintain stimulation within a predefined therapeutic window [18]. This physiologically responsive mechanism likely contributed to the sustained improvements observed in this patient. This stability may be due to the use of ECAPs' biomarker, measuring neural recruitment in real-time. The ECAPs’ amplitude reflects the number of dorsal column fibers activated at each stimulation pulse; for example, a stronger stimulus sensation can be achieved in patients with a larger ECAP. The system automatically adjusted its output to maintain a stable therapeutic window [18].

Despite higher initial costs, closed-loop SCS may offer long-term and effective clinical outcomes. Studies suggest that improved therapeutic success rates and reduced reliance on adjunctive therapies can result in better outcomes over time [19]. Although promising outcomes were observed, it is still important to interpret this case within the context of its inherent limitations. A single case report cannot determine generalizability, and individual variability in response to neuromodulation must be considered.

## Conclusions

ECAP-controlled closed-loop SCS provide a significant advantage in the management of refractory lumbar radiculopathy, including meaningful pain reduction and functional improvement, which ultimately enhances overall quality of life. The ability to deliver real-time, physiologically responsive stimulation represents an important advancement in personalized pain management.
